# A Compelling Health Promoting Primary Care Clinic Using a Settings-Based Approach: A Demonstration Project

**DOI:** 10.1177/15598276241303728

**Published:** 2024-12-01

**Authors:** Mary Larson

**Affiliations:** 1 Department of Public Health, 3323 North Dakota State University (NDSU), Fargo, ND, USA

**Keywords:** primary care, health promotion, lifestyle medicine, settings-based approach

## Abstract

The Ottawa Charter for Health Promotion, issued by the World Health Organization in 1986, called for several strategies to promote the public’s health. One of the strategies was to create health-promoting health services. Over 35 years have passed since the Ottawa Charter was released, since then, efforts to improve health care have been implemented such as the Patient-Centered Medical Home, the Triple Aim, and the Affordable Care Act, yet little has been done to reorient the cultural and physical environment of health care services to one focused on health promotion. In this perspective, the author offers a compelling description of how one primary care clinic, serving an ethnically and economically diverse population, utilized a settings-based approach to design and implement several health-promoting policies, systems, and environmental strategies. This reorientation of a primary care clinic to one that is health-promoting leveraged the clinical specialty of lifestyle medicine as a cornerstone of the settings-based approach.

“Primary care clinics adopting a settings-based approach should implement a comprehensive data collection strategy.”

More than 35 years ago members of the World Health Organization (WHO) issued the Ottawa Charter for Health Promotion (1986).^1^ The purpose of this international appeal for action was to outline and advocate for basic health promotion strategies to be used by any entity that assumed responsibility for public health. The document identified five important directives. They are, “build healthy public policy,” “create supportive environments,” “strengthen community action,” “develop personal skills,” and “reorient health services.^2^” Unfortunately, the Ottawa Charter’s call for a more holistic take on health promotion was ignored. Yet, soon after issuing the Ottawa Charter, WHO began the International Network of Health Promoting Hospitals and Health Services (HPH), a non-profit, non-governmental organization that also relies on the settings approach to health promotion.^3^ Both set out to address health care organizations’ structure and function with the goal of creating environments that no longer focused on disease diagnosis, and treatment, but rather the promotion of health and human flourishing.

While government and non-profit health organizations have certainly had some impact on the American health service industry (e.g., American Academy of Pediatrics’ Standards of Child Health Care [medical home concept^4^], Institute for Health care Improvement [Triple Aim^5^], the Affordable Care Act,^6^ etc.), these efforts have yet to create an overarching culture of health promotion within primary care organizations. Additional changes to clinical practice have also occurred with the growth and development of lifestyle medicine (LM)^7,8^; exercise as an approach to managing chronic health conditions^9,10^; the integration of screening and other interventions that promote health enhancing behaviors^11,12^; patient activation^13^; shared decision making^14,15^; and increased screening and support of social determinants of health.^16–18^ Each of these approaches provides valuable tools in the effort to achieve the goals of the Institute for Health care Improvement’s Triple Aim, which include improving patient care, enriching patient health, and lowering health care costs.^5^ Still, most of these initiatives end up being applied to behavioral causes late in the provision of health care, especially in terms of many prevalent non-communicable diseases that cause morbidity and early mortality today.

The purpose of this project was to utilize evidence-based strategies to further encourage and enhance the patient-centered focus of a primary care clinic^19–21^; improve patient health by focusing on factors that contribute to primary and secondary prevention^22,23^; and ultimately, improve the quality of patient care.^24–26^ This article describes a demonstration project that was set up at a local clinic in a mid-sized, Midwestern community that served culturally and ethnically diverse patients who relied upon a wide variety of payer sources. The project was developed in an attempt at improving outpatient health-promoting services for individuals, including those who were under- or uninsured, and seeking care at a federally qualified health clinic (FQHC). By describing this project, this author hopes to amplify and provide further support for the reorientation of health services to health promotion and disease prevention.

It is especially important to note the problematic nature of improving federal and state-level policies so that an increase in health care *access* might occur, while at the same time ignoring *how* and in what *environment* that care is provided.^27–30^ By not attending to the environment in which health care is provided, stakeholders (health care administrators and other professionals, patients and payors of all types, etc.) will find it almost impossible to obtain Triple Aim results. It is imperative that we improve the *culture of health*^31,32^ within care facilities, beginning with those organizations serving the largest number of patients, primary care clinics. In an effort at reorienting health care services in a non-hospital based primary care clinic, the author offers one model of how to leverage a settings-based approach to develop a *health-promoting primary care clinic*.

## Settings-Based Approach

In an attempt at refocusing a local FQHC on patient health promotion and disease prevention, this author led a multi-year, comprehensive project dedicated to influencing organizational policies, systems, and environmental structures.^33–35^ The primary impetus for this work came from the multi-ethnic patients who served on the clinic’s Patient Advisory Council (PAC). The PAC was established a couple of years before the planning for the new building commenced. These individuals asked for improvements in the quality of patient care services. Specific concern was expressed for patients who suffered with health disparities related to race, ethnicity, and socioeconomic status.^36,37^ After many conversations with this group, our project’s main questions centered around how we might best create clinical policies, systems, and an environment that promotes patient health for all.

Because members of a local FQHC’s leadership had already begun planning the renovation of a historic building in a way that would double its patient care capacity, our efforts at creating a health-promoting clinical environment evolved over time. Soon, the leadership team consisting of the Chief Executive Officer (CEO), Chief Financial Officer, Medical Director, and Clinical and Quality Improvement Manager in collaboration with clinical staff and practitioners began to consider adding patient-centered programs and services funded by a capital campaign, including supplemental funding from the Health Resources and Services Administration. At the time of this expansion, the author, who served as a Lifestyle Medicine Provider and Employee Wellness Committee Chair, was invited by the CEO to meet with architects and advocate on behalf of the Employee Wellness Committee for constructing an environment focused on health promotion and disease prevention. Despite the challenges faced in these meetings, significant progress was made. The author, along with key community stakeholders such as local exercise scientists, public health nutritionists, and health promotion specialists, worked hard to successfully reframe the concept of using clinical space for health promotion. We presented these ideas as *clinical* strategies and aligned them with lifestyle-based medical approaches. As a result of having engaged in this approach, the clinic: dedicated physical space to a LM department; adapted organizational policy and systems; elected to implement specific LM forms within the electronic health records (EHR); increased its collaboration with a local YMCA and AmeriCorps volunteers; and provided health promotion training for staff.

## Collaborative Achievements

The clinic adopted the patient-centered medical home model prior to designing the new clinic and the Patient Advisory Council (PAC)^38^ was established as a result. It was important that this group of patients be educated about and take part in discussions related to the health promotion refocus of their clinic. Fortunately, the author served as a staff liaison to the PAC and was able to introduce this change in focus by asking PAC members what they wanted to see in an improved, redesigned clinic. The group responded by reporting their need for a physical space dedicated to LM, a teaching, demonstration, and learning kitchen [Photograph 1], a fitness room [Photograph 2], and two small locker rooms, each with a shower. While the author supported this vision, it was initially met with mixed reactions by architects and clinic leaders. Such features are not routinely part of a contemporary primary care clinic setting and are rarely, if ever offered free of charge to all patients. Therefore, the author had to work hard to communicate our innovative approach to patient health care; an approach that went well beyond traditional medication-based and fee-for-service treatment.

**Photography 1.**
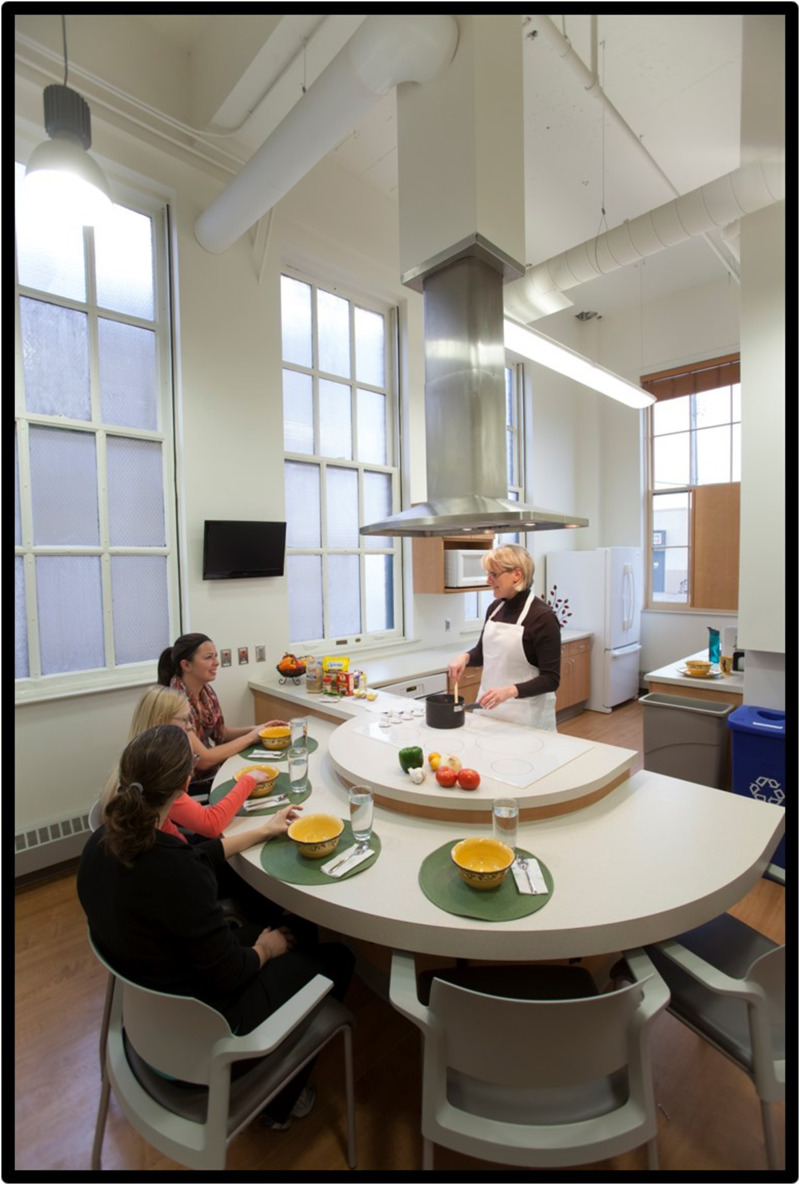
Teaching Kitchen.

**Photography 2.**
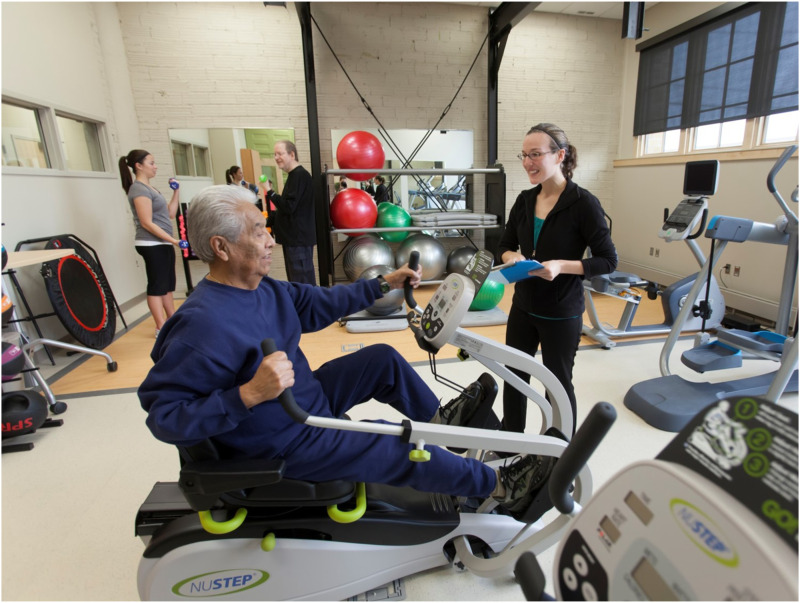
Fitness Room.

The employee wellness committee, which was formed prior to the renovation of the clinic, established an organizational policy that provides vending guidelines requiring healthy food and beverage selections. Most national vending brands were not able to meet these requirements. As a result, the employee wellness committee agreed to stock the clinic’s vending machine with food and beverages that meet the newly established criteria.^39,40^ We identified appropriate vending products and purchased a refrigerated machine for patients and employees using grant funds [Photograph 3]. Discussions around vending machine products soon expanded to include food and nutrition policy statements regarding all provisions purchased by the clinic, including the food and drink being offered at meetings. Nutritional criteria were developed by a dietetic intern under the supervision of the author and revised until approved by the employee wellness committee. Staff training for those who typically order food for meetings was provided, along with a list of catering options with sample menus to help guide healthy selections.

**Photography 3.**
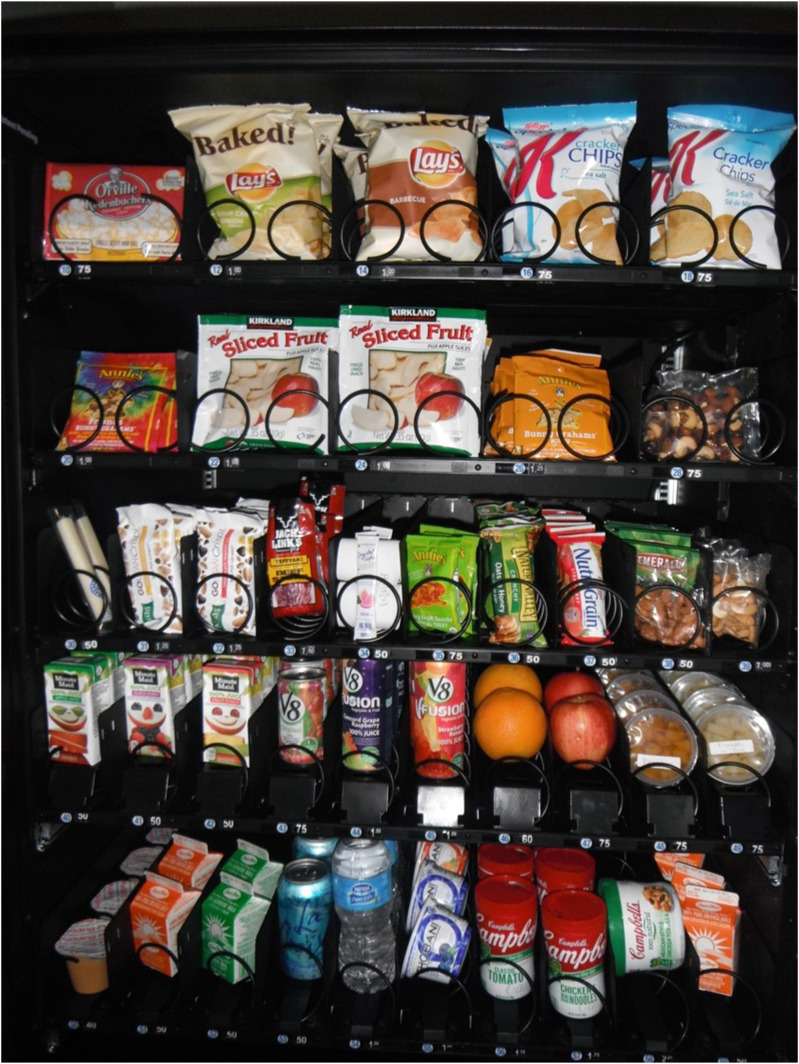
Non-Profit Vending.

**Figure 4.**
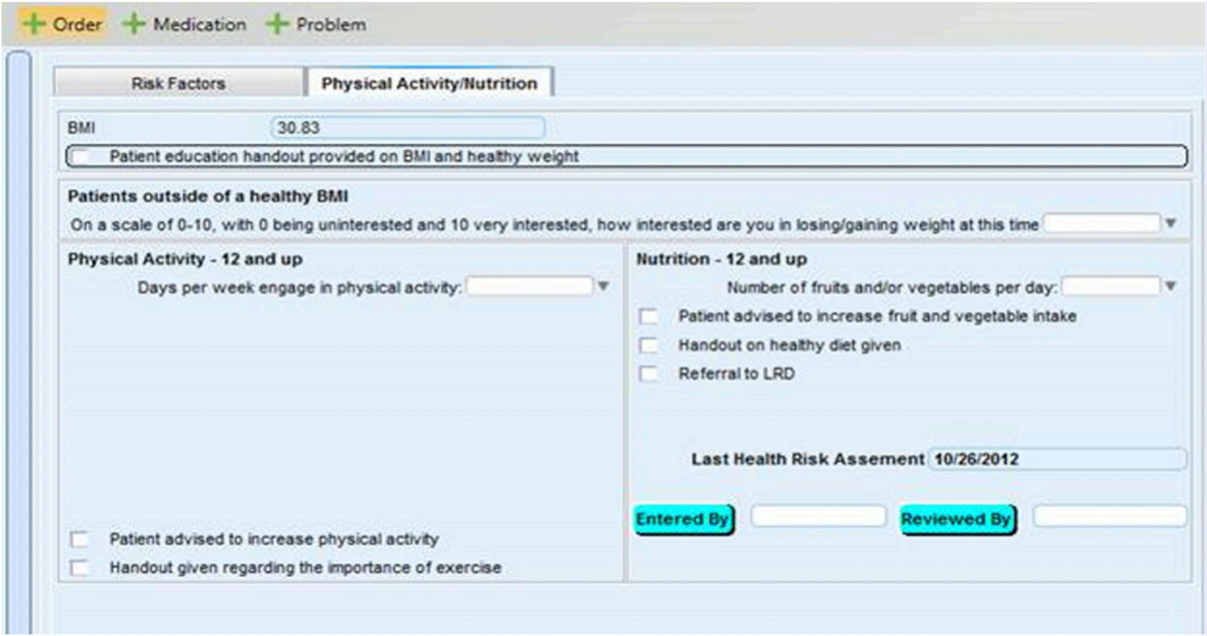
Physical Activity and Nutrition Vital Signs.

**Figure 5.**
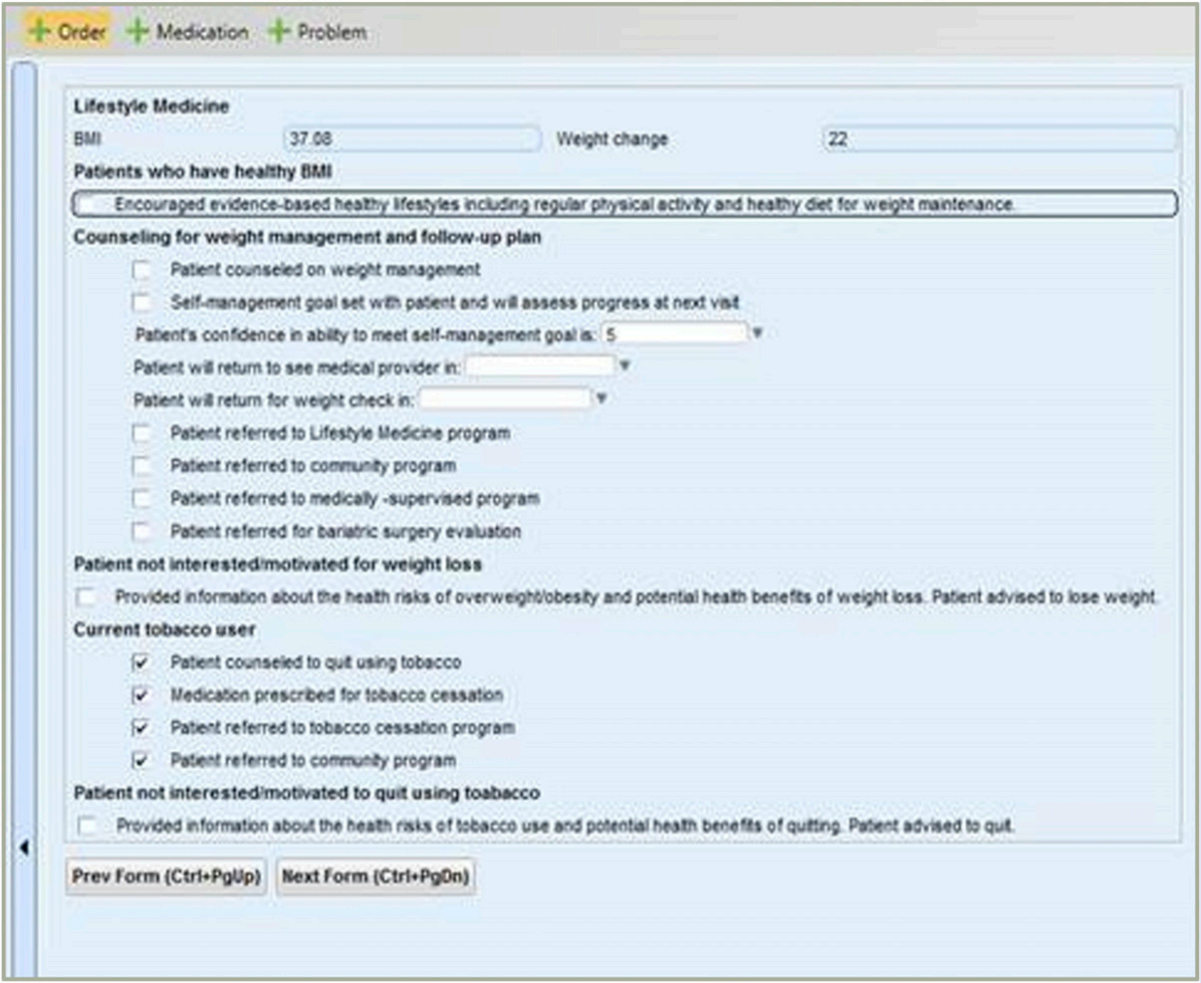
Lifestyle Medicine Plan.

A team of clinic staff, including the Clinical and Quality Improvement Manager, Nurse Manager, Medical Director, and author, developed several behavioral assessment questions that were embedded in the clinic’s EHR intake screens [Figure 4]. Additional questions concerning frequency/duration of physical activity and the consumption of fruits and of vegetables were also added.^41–44^ These questions were followed by informational prompts that encouraged patients to increase their physical activity, improve their fruit and vegetable intake, or take advantage of a LM referral. Additionally, an EHR LM plan screen [Figure 5] was developed. The clinic’s Medical Director eventually determined that this screen should appear first to communicate its importance to both patients and health professionals. The series of screens helped facilitate patient-centered goal setting and referrals to members of the LM team.^45,46^ Motivational interview (MI) training^47–49^ and other professional learning opportunities were delivered to nurses and providers. Training was centered around topics such as health literacy,^50,51^ self-management goal setting,^48^ patient activation,^13^ and health coaching.^52,53^ Training of this kind helped increase patient-centered care and intentionally integrated LM into the care culture of this clinic.

Two additional organizational collaborations are worth noting regarding this project. Prior to the FQHC renovation, the author worked with a local YMCA to negotiate access to a sliding fee scale (SFS) that could be applied to health club memberships for eligible patients. Through this partnership the author screened and qualified patients for the sliding scale and further streamlined this process, thereby reducing barriers for patients seeking access to community wellness and physical fitness facilities. Eventually, the author learned that this YMCA also housed the regional AmeriCorps volunteer program. Upon meeting with the AmeriCorps Program Director, a request was made to utilize AmeriCorps volunteers in the clinic’s LM department as health coaches. During the first year of this collaboration, the LM department introduced a single AmeriCorps volunteer. The following year, two volunteers joined the department and in the third year it hosted five AmeriCorps volunteers. Each volunteer was responsible for working 20 hours a week, given a specific job description, and trained as a health coach. In addition to helping patients with self-management goals, volunteers were also responsible for fulfilling duties related to other LM programs and services [Photograph 2]. Because the AmeriCorps program was involved with and supported this collaboration, clinic costs for volunteer services were minimal.

This project relied on a strong partnership that was well-developed prior to the clinic’s renovation project with a state health department health improvement project. The statewide health improvement project provided financial assistance through grants that helped purchase equipment for the teaching, demonstration, and learning kitchen and further supported other health promotion programming and services. They also provided supportive coaching and goal setting assistance to clinical staff as a means of helping them maintain numerous health-promoting services.

## Lifestyle Medicine Growth

Between 2010 and 2012, LM staffing had increased from 1.3 FTE to 5 FTE. Health-promoting programs and services were increasing and being actively offered through the LM department. Programming included clinical nutrition appointments for individuals and groups; strength and conditioning training; prevention and management sessions for people with chronic health conditions; tobacco cessation clinics; and classes to learn about cooking for health and healing. The LM department offered a weekly *Nutrition on the Move* program with demonstrations held in the patient waiting area [Photograph 6]. This program regularly featured fruits and vegetables that were locally in-season and was a favorite among patients and staff. Another offering was designed to increase a patient’s physical movement while they were waiting to see their care provider. Patients could either use a floor-pedal machine or visit the fitness room while waiting to be called to attend their appointment.

**Photography 6.**
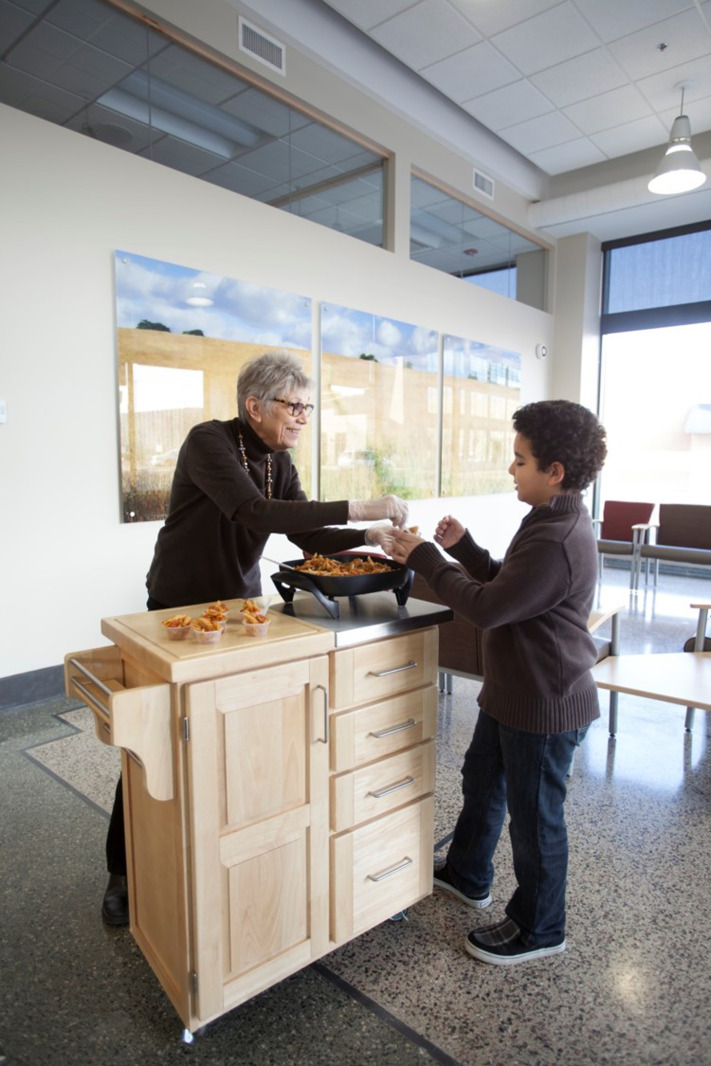
Nutrition on the Move.

## Healthy Built Environments

The scarcity of publications describing health-promoting primary care clinics, such as the one offered by this paper, is an indicator of how far behind the U.S. has been when it comes to adapting policies, systems, and environments to focus on overall health promotion as opposed to medicine-based treatment. This demonstration project provides one example for how clinics might weave together older, but critically important calls for change as outlined by the Ottawa Charter, with more recent recommendations from organizations such as HPH and the Centers for Medicaid and Medicare.

Environmental changes alone will not be enough to reorient the entire American health system to one that is focused on health promotion. Still, paying attention to the policies, systems, and environment within primary care clinics is one essential step toward progressing health care organizations to a point where people and communities have more control over key factors that influence their health.^54^ Other changes need to be made as well. For example, experts note that the two most powerful lifestyle behaviors for preventing, treating, and even reversing the leading chronic health conditions amongst Americans (eating healthy and physical activity) are health promotion tactics that require intentional screening and integration into health care clinics.^11,55^

Buildings designed as health-promoting facilities are built by people who understand the importance of common environmental features such as temperature, lighting, and air quality. These environments also include spaces that improve the equity and access to health care using strategies such as culturally relevant signage, healthy food promotion, and positive social interactions.^27,28,30,56^ Improved environmental changes such as these, combined with newly formulated clinical policies and systems, can markedly ease health behavior change activities for patients.

Additionally, behavioral change or “adaptive work” can be further aided by health care professionals with the knowledge, skills, and abilities to engage in comprehensive, positive, and productive patient interactions.^57^ Unintentional but real harm can be caused when providers rely solely on technical solutions (e.g., medications or other medical procedures) to treat some health issues. In these instances, harm can be categorized by degree. The first degree of harm might be negative side-effects which occur due to a particular medication or treatment. The second degree of harm could result if a patient simply gives up on or dismisses important adaptive behavior work due to their confidence in a medication or procedure. The third degree of harm is from the complacency experienced by many health care professionals about their adaptive work to learn about and use powerful LM treatments and the evidence-based methods (e.g., MI) to help their patients make and sustain behavior change.^57^ Skillful application of evidence-based, adaptive solutions such as motivational interviewing^48,49^ can be game-changers.

Health care professionals are more likely to engage in productive lifestyle behavior conversations with their patients when supportive policies and systems are embedded within their clinic’s environment.^11,55^ Additionally, health care professionals must have adequate LM training and understand behavior change methodologies in order to help their patients’ when challenging adaptive work is needed.^12,58,59^ Unfortunately, many of today’s health care professionals have not been trained to effectively use LM or other evidence-based behavior change strategies. This situation represents a severe educational gap within our current health care workforce. It is, therefore, critically important that clinic administrators and other health care leaders acknowledge the need for professional development as these organizations move toward a system that is more keenly focused on health promotion and disease prevention.

## Lessons Learned

(1) It is important to provide leadership, including the CEO, Clinical Manager, and Medical Director, with a clear and concrete explanation of how a settings-based approach will be implemented and experienced in practice.
(2) Integrating Lifestyle Medicine into primary care through a settings-based approach offers an effective method to transform clinics into more health-promoting environments.
(3) To successfully reorient a clinic, it is essential to incorporate updated clinical policies, streamlined systems, and comprehensive staff training into the transformation plan. This integration ensures that all team members understand and feel confident in their roles during the implementation process.
(4) Primary care clinics adopting a settings-based approach should implement a comprehensive data collection strategy. This strategy should track several key metrics: the degree to which the lifestyle factors are assessed using the new EHR forms, the frequency of patient referrals to newly introduced programs and services, how often patients utilize clinical enhancements, changes in clinical outcomes, and the extent to which staff make use of the improved facilities. Gathering this diverse range of data will provide valuable insights into the effectiveness and impact of the new approach Table 1.

**Table 1.** Health-Promoting Clinic: Settings-Based Strategies Logic Model.

Policy, System, Environment	Health-promoting strategy	Logic of change
Organizational, person-centered strategies	The PAC, established as part of the patient-centered medical home criteria, influenced environmental changes to the clinic by promoting health-related issues that they felt were especially important	If we want people (patients, staff, community organizations, etc.) to engage in and advocate for innovation, then we need to actively include them as full partners in the design processes that will help clinics reorientate to a broader philosophy of improving the health and overall well-being of those who are served
	The employee wellness committee supported healthy vending machine choices and nutritious food and drink being always served throughout the clinic	
	Health care staff took part in professional development: Exercise is medicine; food is medicine; LM; behavior change methodologies; motivational interviewing; health literacy; patient self-management; patient activation; and health coaching	Health care professionals need additional training to develop the knowledge, skills, and abilities to successfully implement health-promoting policies and systems
	Clinical staff helped integrate health promotion into the EHR forms and facilitated their implementation	
	Community partners aligned with the clinic to provide access to supplies, equipment, and a source of low-cost personnel	
Organizational built environment strategies	Establish physical space for LM	If the goal is to reorient health services, we should consider starting with primary care organizations. When TA is our goal, we must commit to providing health care in a unique way. This includes creating an environment dedicated to health-promotion and disease prevention If LM is an important set of knowledge, skills, and tools for improving patient care, patient health, and reducing costs, then there should be dedicated spaces for the specialists using it
	Establish a physical space for a teaching, demonstration, and learning kitchen	If food is medicine, then a kitchen area is an important space to include in a primary care setting so patients (and staff) may receive cooking and nutritional training
	Establish a physical space for deconditioned individuals to engage in physical movement	If exercise is medicine, then primary care clinics should consider making space to engage patients in physical activity that is overseen by health coaches. Engaging in exercise is intimidating for patients who are deconditioned
Organizational policy strategies	Develop dietary guidelines for food purchased by the clinic and made available to patients and staff	Food that is made available to patients, staff, and visitors when they are in health care organizations sends a message about how seriously clinic staff takes health promotion. Gone are the days (at least they should be) that someone will find a cupcake kiosk in their local clinic
Organizational settings and systems strategies	Implement EHR forms that assess and promote dietary, as well as physical activity health indicators	If the objective is to create a health-promoting clinic, then it is imperative to consider every component of that clinic’s environment—built, physical, social, interpersonal, emotional, and electronic—from the time a patient approaches, enters, and remains in the clinic, until the time they leave
	Purchase vending machine and stock with food and drinks that meet nutritional criteria	
	Collaborate with local organizations that have shared mission and values	
	Identify partners who will fund initiatives	
	Form and sustain an employee wellness committee that is comprised of one person from each department	
	Adopt cost-effective methods to extend health-promoting services within the clinic	
	Create methods of actively engaging patients and staff in health promotion efforts	
	Communicate mission and purpose with passion to leadership	
